# The continuing search for physiologic pacing

**DOI:** 10.18632/aging.101928

**Published:** 2019-04-27

**Authors:** Dominik Beer, Faiz A. Subzposh, Pugazhendhi Vijayaraman

**Affiliations:** 1 Division of Cardiac Electrophysiology, Geisinger Heart Institute, Geisinger Commonwealth School of Medicine, Wilkes-Barre, PA 18711, USA

**Keywords:** His bundle pacing, physiologic pacing, right ventricular pacing, dyssynchrony

Since the early 1960s, cardiac pacing has been the definitive therapy for bradycardias. Beginning with single chamber pacemakers and the subsequent advent of atrioventricular (AV) sequential pacing addressed AV dyssynchrony but inter-and intraventricular electromechanical dyssynchrony remained. The problems associated with right ventricular pacing (RVP), particularly when pacing burden is high, have been demonstrated in several large trials and include an increase in the burden of atrial fibrillation (AF), heart failure admission, and mortality [1,2]. Cardiac resynchronization therapy (CRT) was developed to promote physiologic biventricular activation and this technology has certainly improved heart failure outcomes and mortality in those patients with severe left ventricular dysfunction and native left bundle branch block physiology. However, the role for CRT in patients with bradyarrhythmias without heart failure is unclear. Furthermore, optimal left ventricular lead placement may not always be technically possible and certain patient population may not respond in a desirable fashion. The need for cardiac pacing continues to rise as our population ages and the decades-long quest for an optimal ventricular pacing site remains one of electrophysiology’s biggest challenges. His bundle pacing (HBP) is an alternate technique designed to promote physiologic ventricular activation by depolarizing the native ventricular circuitry at the bundle of His before it subdivides into the left and right fascicles [3].

While several studies have demonstrated the feasibility of resynchronization with HBP and the benefit to hemodynamics when compared with RVP [2], more recent reports have shown a benefit in clinical and echocardiographic outcomes. Abdelrahman et al. [4] recently compared RVP and HBP in the largest-to-date observational cohort of consecutive patients (332 HBP vs 433 RVP). They showed that HBP is more feasible than initially reported with a 92% success rate, and that it is associated with a significant decrease in the composite endpoint of heart failure hospitalization, upgrade to a CRT device, or death (25% vs 31.6%; HR: 0.71; p=0.02) over a mean follow up of 725 ± 423 days. On subgroup analysis of patients with a ≥20% ventricular pacing burden, the margin between HBP and RVP widened further (25.3% vs 35.6%; HR: 0.65; p=0.02) while there was no statistically significant difference in outcomes in those patients with a pacing burden <20%. The primary endpoint was driven by a difference in heart failure admissions between groups but there was a trend toward reduced mortality in the HBP group as well (17.2% vs 21.4%; p = 0.06). One might extrapolate that the patients with the most to gain from physiologic pacing is the population in which a high burden of pacing is unavoidable. The incidence of pacing induced cardiomyopathy was observed in 22% of patients with RVP compared to 2% in the HBP group during long-term follow-up [1]. Interestingly, only one-fourth of these patients underwent upgrade to biventricular pacing. In other studies of RVP, pacing induced cardiomyopathy was observed in 12.5-19.8% of patients [5, 6]. Despite significant improvement in LVEF with CRT in patients with pacing induced cardiomyopathy, upgrade to CRT was performed in only 28% of these patients [5,6]. It is likely that many elderly patients with pacing induced cardiomyopathy are not considered for CRT due to associated co-morbidities or lack of awareness. Therefore, it is even more important to prevent pacing induced cardiomyopathy by investing in more physiologic pacing such as HBP. Recent cohort data [7] reported a 95% success rate of HBP implantation in patients undergoing AV node ablation as well as significant improvement in the LVEF and NYHA functional class.

It is necessary to mention, as with any new technology, that there are some shortcomings and growing-pains of HBP. In the recent study by Abdelrahman et al. [4], the mean procedure and fluoroscopy times were longer with HBP (70 ± 34 min vs 55 ± 25 min; p<0.01 and 10 ± 7 vs 7 ± 5 min; p<0.01 respectively) and higher His lead capture thresholds compared with RV capture thresholds on implantation (1.3 ± 0.85 V at 0.79 ms vs 0.59 ± 0.42 V at 0.5 ms, respectively p<0.01) and on 24 month follow up. This translates to shorter battery life and likely more frequent generator changes. In the HBP, 4.6% of patients required lead revisions due to failure to capture or unacceptably high capture thresholds compared to 0.5% in the RVP group [2]. However, in the short time since its conception, HBP has seen an improvement in success rates, thresholds, lead stability as technology, technique, and experience have improved.

Further investigation in the form of well-crafted randomized, controlled trials is needed to determine the role of HBP and whether the debate on the optimal ventricular pacing site can be closed [8]; however, the shortcomings of HBP will undoubtedly fade as battery, lead, and lead delivery technology advance. Particularly in an aging population where significant co-morbidities are more prevalent, maintenance of not only AV but also VV synchrony to reduce the incidence of heart failure hospitalization, cardiomyopathy, burden of atrial fibrillation, and potentially reducing mortality is a worthy endeavor.
